# How I do it—the posterior question mark incision for decompressive hemicraniectomy

**DOI:** 10.1007/s00701-021-04812-4

**Published:** 2021-03-31

**Authors:** Michael Veldeman, Mathias Geiger, Hans Clusmann

**Affiliations:** Department of Neurosurgery, RWTH Aachen University Hospital, Pauwelsstrasse 30, 52074 Aachen, Germany

**Keywords:** Decompressive hemicraniectomy, Posterior question mark incision, Incision type, Surgical site infection, Cranioplasty, Skalp vascularization

## Abstract

**Background:**

Decompressive hemicraniectomy (DHC) is a lifesaving procedure which every neurosurgeon should master early on. As indications for the procedure are growing, the number of patients eventually requiring skull reconstruction via cranioplasty also increases. The posterior question mark incision is a straightforward alternative to the classic trauma-flap and can easily be adopted. Some particularities exist one should consider beforehand and are discussed here in detail.

**Methods:**

Surgical steps, aids, and pitfalls are comprehensively discussed to prepare surgeons who wish to gain experience with this type of incision.

**Conclusion:**

Due to the lower complication rate after cranioplasty, the posterior question mark incision has superseded the traditional pre-auricular starting anterior question mark incisions, in our department for the performance of decompressive hemicraniectomies.

**Supplementary Information:**

The online version contains supplementary material available at 10.1007/s00701-021-04812-4.

## Relevant surgical anatomy

The scalp is the only integument supplied solely by direct cutaneous arteries without contribution of deep perforators [5]. A rich and well-lined anastomotic network between the three main supplying externa carotid artery branches compensates for this shortcoming. Although transcalvarian supply of the skin via meningeal internal carotid artery (ICA) branches is described, its role remains negligible. Also, the anastomosis of the ophthalmic end branches of ICA with the facial artery do not play a major role under physiologic circumstances [4]. The trauma-flap or reversed question mark incision is the largest scalp flap dissected in routine neurosurgical practice. The incision requires sacrificing the post-auricular and sometimes at least part of the superficial temporal artery (STA). This leaves the facial artery and its terminal branches including ICA supported ophthalmic vessels, as the primary arterial supply to the flap. However, by placing the beginning of the incision behind the ear, the vascular pedicle is broadened significantly and the STA can be preserved in its entirety.

In our experience, the reduced vascular supply after the traditional anterior question mark incision does not contribute to increased complications after primary DHC surgery. Secondary cranioplasty is required not only for protective and cosmetic purposes, but also supports neurological recovery. This treatment is still plagued by a relative high complication rate [3]. In a recent analysis of 186 DCH patients, the infectious complications rate after secondary cranioplasty was reduced by 14.4% in patients operated via the posterior incision type [6]. We believe that for the healing of a large avascular bone flap, optimal arterial supply is insufficiently provided after the classical trauma-flap incision.

**Fig. 1:**
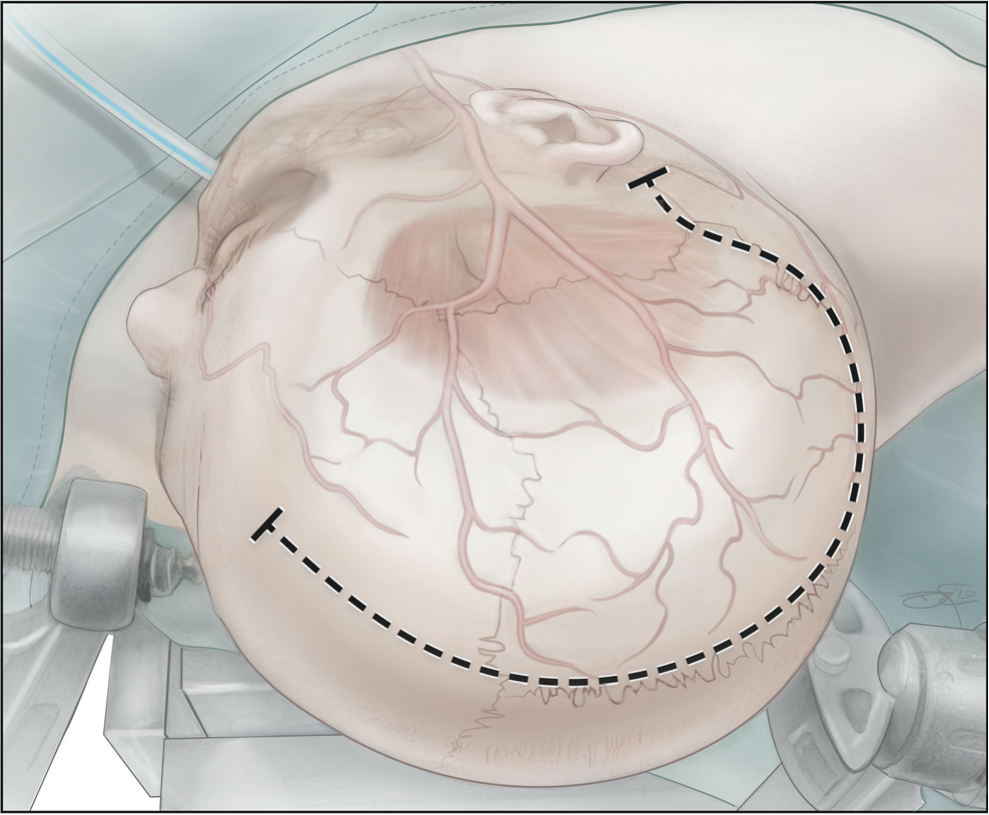
Illustration of correct positioning for a right-sided decompressive hemicraniectomy. The patient is positioned in the supine position with padding of the ipsilateral shoulder to achieve an effortless head rotation of 90°. The incision starts two finger breadths posterior to and at the level of the mastoid base and is carried in the direction to the inion to reach the midline. The incision then follows the midline up to widow’s peak

Relevant palpable bony anatomical landmarks are the external auditory canal, mastoid process, mastoid notch the inion, and widow’s peak depicted in Fig. 1.

## Description of the technique

The severity of the underlying disease usually turns a DHC into an emergency procedure. In order to minimize time-loss and achieve optimal efficiency the whole operating room team, e.g., assistant, anesthesiologist, and scrub nurse need to be familiar with this “DHC drill.”

The patient is positioned supine with padding under the ipsilateral shoulder and pelvis to allow 90° rotation of the head without excessive torque on the cervical spine enabling optimal venous outflow. The head piece of the OR table can be moderately elevated to improve venous outfolw, but not excessively to avoid downward traction on the cervical spine. We recommend a 3-point fixation in a Mayfield head holder or similar system for optimal skull immobilization during the craniotomy and to increase working space around the patients head. To save time in emergencies, in experienced hands, the incision can simply be marked with a clipper during shaving. The midline is identified at widow’s peak and hair removed one clippers width, along the midline in one smooth motion until reaching the inion. Here, the incision bends behind the ear to end at the level of the base of the mastoid, about two finger breadths posteriorly of the mastoid notch. Standard prep and draping techniques are used.

**Fig. 2:**
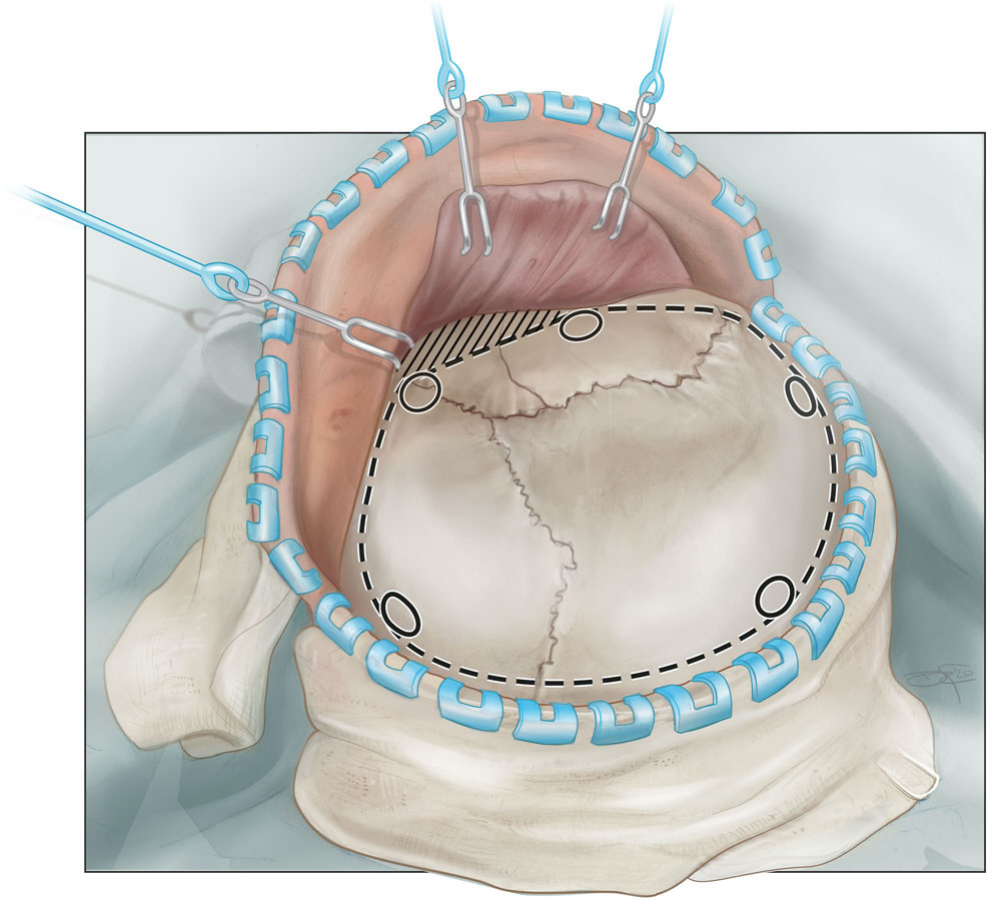
Illustration of bony skull landmarks. Special attention should be paid to identify the sagittal and coronal sutures as well as the pterion and lambdoid suture. Depending in individual skull morphology, retracting of the flap caudally to expose temporal and sphenoidal bone may be more challenging compared to the classic question mark incision. Occasionally, larger parts of the skull will need to be rongeured off (area marked by diagonal lines). We typically place the pterional and temporal burr holes first, followed by the paramedian burr holes starting frontally and ending with the one behind the lambdoid suture.

The incision should start behind the mastoid notch and is not to be carried below the tip of the mastoid process to avoid damaging the occipital artery. Just as when preserving the STA in a classical question mark incision, the initial cut should not be deeper than the hypodermic layer in which the occipital artery travels. In a second step, the occipital artery is identified by blunt spreading of tissue with scissors and preserved, after which the incision is driven deeper anterior from the vessel (Figs. 1 and 2).

**Fig. 3:**
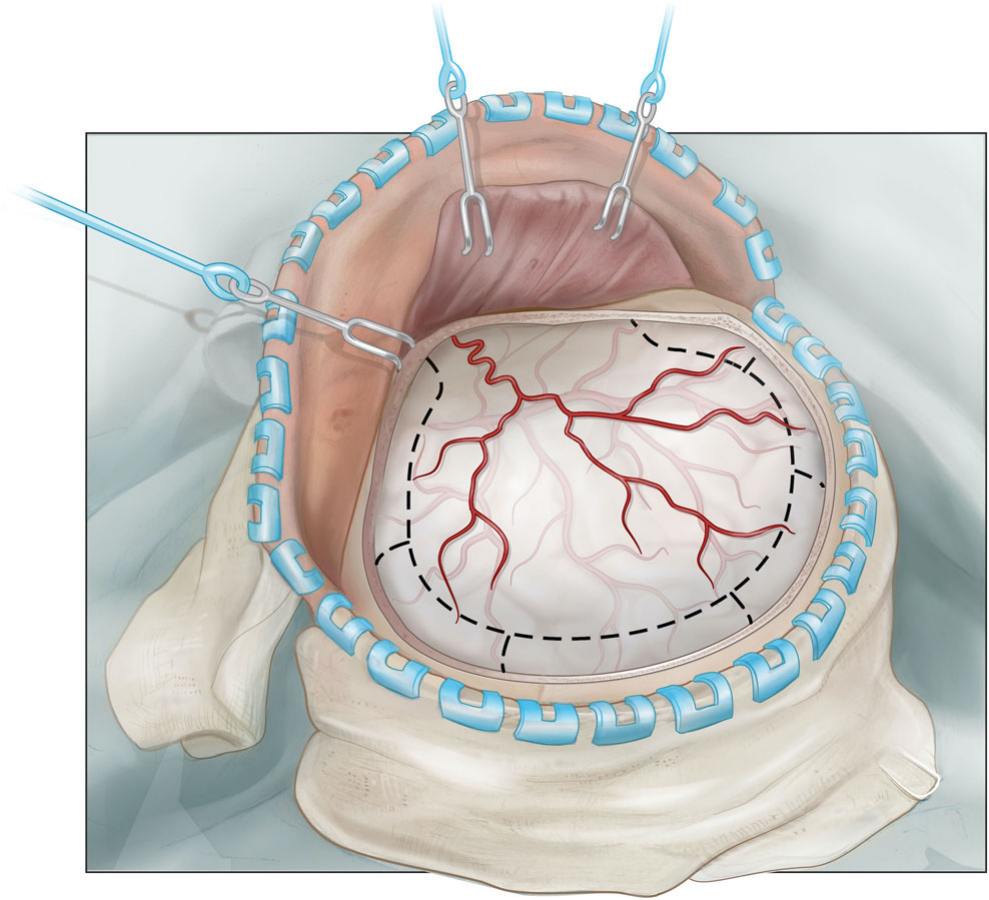
Illustration of the planned durotomy. Depending on the urgency of the indication, special attention can be paid to achieve hemostasis before dural opening. The dura can be opened in a stellate fashion but more often we apply a U-shaped flap incision instead, allowing the preservation of a broader vascular pedicle. Centrifugal tension-relief-cuts are placed at 3–4 cm distance apart and carried up to the bony rim

**Fig. 4:**
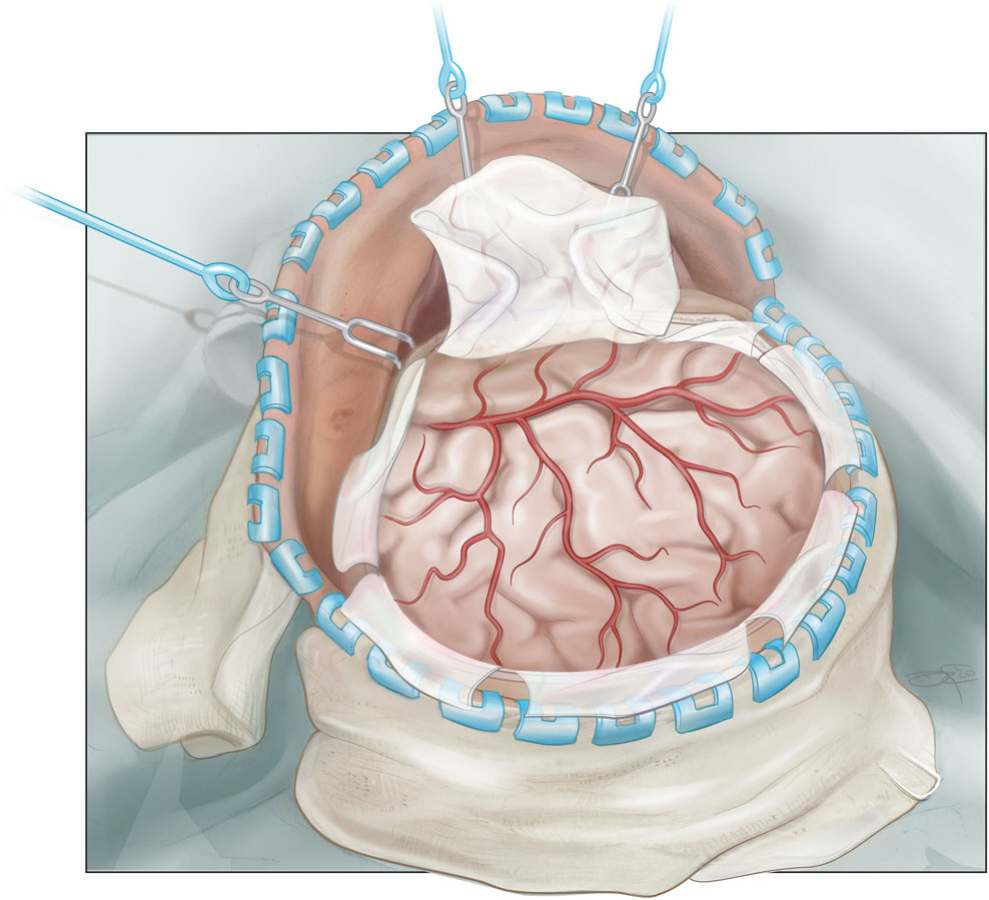
Illustration of the final result after durotomy. We apply a rapid closure technique in which the dura is simply draped over the brain surface without the application of an expansion duroplasty

The further steps do not deviate relevantly from a standard trauma-flap procedure. The skin flap is bluntly dissected, folded over and retracted with flexible hooks. Care is taken to incorporating the outer layer of the temporal fascia into the skin flap, not to damage frontal branches of the facial nerve. The temporal muscle is either dissected with monopolar cautery or a cobb elevator. Five burr holes are placed: frontal, parietal, key hole, posterior temporal squama, and lambdoid suture. After dural elevation, burr holes are connected with a craniotome. If proven too thick, the minor sphenoid wing can be thinned out with a cutting drill and broken (Figs. 3 and 4). Temporal squama is rongeured off till the level of the temporal base is reached from the sphenoid wing anteriorly to the petrosal bone. The dura can be incised in a stellated or steeled fashion. A rapid closure technique is applied without duraplasty [2, 7]. Usually, two non-suction drains are left behind in the epidural space. After subcutaneous closure, the skin is stapled and the wounds is betadine draped and dressed.

## Indications

The spectrum of indications of this altered incision DHC is the same as a standard decompressive hemicraniectomy. A discussion of the indications for decompressive hemicraniectomy is beyond the scope of this technical report.

## Limitations

There are three main drawbacks to this posterior type of skin incision. Firstly, downward folding of the skin flap is somewhat hampered by the ear. Depending on individual anatomy, this sometimes limits the exposure of the temporal bone in comparison to a classic trauma-flap. Care has to be taken not to dissect to caudally at the level of the petrosal bone, in order not to damage the external auditory canal. However, as the anterior temporal bone is rongeured off, the somewhat limited outer exposure does not prevent optimal decompression of the temporal region. Secondly, if a completed 90° head rotation cannot be achieved, the post-auricular area can be harder to reach. If the patient is fixed in a pin head-holder and enough working space is provided, surgeon’s flexibility can compensate for this drawback. And finally, because the skin flap rotates downwards around a line parallel to the skull baseline and not ventrocaudally as in a classical trauma-flap, this reduces the downward working area. In reality, this is not a real drawback, just something to get used to.

## How to avoid complications

The main complications after DHC are post-operative hemorrhages, surgical site infections, and general wound healing problems with or without CSF leakage [1]. Meticulous hemostasis with special attention to the temporobasal region and the inner face of the temporal muscle should be carried out. If damaged, the middle meningeal artery should be carefully coagulated to avoid large hematomas. Relieving pressure to the wound at the occiput during ICU stay is mandatory by providing padded specialized cushioning and frequently changes of the position of the head. Wounds and bony defect need daily inspection for signs of hematoma or infection.

## Specific perioperative considerations

The urgent nature of the procedure precludes a regular pre-operative workup. However, time should be spent in evaluating coagulation tests and a deranged coagulation function should be corrected appropriately. In case of trauma, cervical imaging should rule out instability before rotation of the head, and the scalp has to be inspected for wounds which should be addressed during the procedure. Finally, special attention should be paid in identifying skull fractures on CT imaging, as this can complicate Mayfield placement, planning of the craniotomy, be indicative for dural tears or might have caused damage to venous sinuses, which can end in major venous bleeding.

## Supplementary Information

ESM 1 (MP4 119037 kb)

## Abbreviations

DHC: Decompressive hemicraniectomy
ICA: Internal carotid artery
STA: Superficial temporal artery
